# Enhanced mechanical and thermal properties of electrically conductive TPNR/GNP nanocomposites assisted with ultrasonication

**DOI:** 10.1371/journal.pone.0222662

**Published:** 2019-09-23

**Authors:** Ruey Shan Chen, Mohd Farid Hakim Mohd Ruf, Dalila Shahdan, Sahrim Ahmad

**Affiliations:** Materials Science Program, Faculty of Science and Technology, Universiti Kebangsaan Malaysia, UKM Bangi, Selangor D.E., Malaysia; University of Maryland Baltimore County, UNITED STATES

## Abstract

Thermoplastic natural rubber (TPNR) was compounded with graphene nanoplatelets (GNP) via ultrasonication and melt blending. The effects of ultrasonication period (1-4 hours) and GNP weight fraction (0.5, 1.0, 1.5 and 2.0 wt.%) on the mechanical, thermal and conductivity properties were investigated. Results showed that the 3 hours of ultrasonic treatment on LNR/GNP gave the greatest improvement in tensile strength of 25.8% (TPNR/GNP nanocomposites) as compared to those without ultrasonication. The TPNR nanocomposites containing 1.5 wt.% GNP exhibited the highest strength (16 MPa for tensile, 14 MPa for flexural and 11 kJm^-2^ for impact) and modulus (556 MPa and 869 MPa for tensile and flexural, respectively). The incorporation of GNP had enhanced the thermal stability. It can be concluded that the GNP had imparted the thermally and electrically conductive nature to the TPNR blend.

## Introduction

In decades, polymer nanocomposites have gained significant attention from many researchers owing to the high potential of these materials in achieving a outstanding property enhancement. This can be achieved by requiring just a small quantity of nanofillers in a polymer matrix as compared to conventional polymer composites containing a significant amount of micron-sized fillers [1]. Among various nanofillers, there is a great interest in low cost graphene for a wide range of graphene based nanocomposites applications such as sensors, batteries and solar cells, anti-static coating, and transparent conductors [2]. Graphene is a two-dimensional (2D) hexagonal honeycomb lattice, one atom thick sp^2^-hybridized monolayer carbon sheet. Not to say it has a larger specific surface area and smaller size than carbon nanotubes, and this structure is advantageous for effectively improving the physical properties of polymers, such as high tensile modulus, high natural mobility and high thermal conductivity [1, 2].

A stack of several graphene monolayers forms graphene nanoplatelets (GNP). In general, the inclusion of small amount of low cost GNP in polymer composites can improve physical, mechanical, conductivity, thermal stability, gas barrier and fire retardancy properties [3, 4]. To a large extent, the dispersion of the GNP embedded in the polymer matrix remarkably affects the improvement of these properties in the resulting composite material. Unfortunately, the high melt viscosity of a polymer and the strong tendency of GNP aggregation result in difficulty in exfoliating GNP as well as in obtaining a uniform and homogeneous dispersion of GNP in a polymeric matrix [5, 6]. In response to this dispersion problem, ultrasonication, mechanical and chemical pretreatments methods have been applied to improve the dispersion of nanofillers in the polymer matrix [7].

The most common dispersion method used for GNP is through sonication, in which the vibrational energy is provided to the nanoparticles for escaping from the surrounding restrained force [7, 8]. Recently, sonication or ultrasound vibration technique has been applied to the preparation of nanocomposites, but with different methods. Covarrubias-Gordillo et al. sonicated a mixture of carbon nanofibers and graphene platelets in gas phase prior to plasma polymerization by propylene to achieve deagglomeration and increase the surface area [9]. Ultrasonic vibrations have been reported to reduce the viscosity of polymer melt, thereby improving the processability of the polyolefin. The clean and efficient ultrasound vibration technology has been applied in the extrusion system by He et al. in order to exfoliate and disperse GNP in polypropylene matrix during melt blending process [5]. Seretis and co-researchers investigated the effect of sonication bath time (20, 40 and 60 min) on epoxy resin/GNP nanocomposites of 1 to 5% GNP contents. The findings showed that in the absence of sonication, the tensile performance increased with the increase of GNP content in the nanocomposites. It is worth to note that during sonication, the GNP layers were more winkled and the ultimate tensile strength of nanocomposites increased with the sonication time [8].

Despite of these studies, the researches on the chemically ultrasonication treatments of pre-mixing nanoparticles with polymeric matrix are still limited. In this present work, the aim was to evaluate the effects of sonication bath time of GNP/LNR pre-mixture and the GNP loadings on the mechanical, thermal, and electrical and thermal conductivity properties of TPNR nanocomposites.

## Experimental

### Raw materials

The thermoplastic resin used was polypropylene (PP) having a density of 0.905 g/cm^3^ and was procured by Propilinas Sdn. Bhd. SMR-L grade natural rubber (NR) was supplied by the Malaysian Rubber Board. The liquid-based natural rubber (LNR) was self-synthesized by photosensitized chemical degradation [10] using the raw solid NR and some chemicals, i.e. methylene blue, rose Bengal and methanol, which were obtained from Sigma Aldrich. The nanofiller used in this study was graphene nanoplatelets (GNP), with a tradename of KNG-150, purchased from KNANO, having a density of 2.25 g/cm^3^, a thickness of 5-15 nm and a diameter of 5 μm.

### Composite preparation

Thermoplastic natural rubber blend and its GNP nanocomposites were compounded via indirect mixing technique using an internal mixer machine (Haake Rheomix 600P). The compounded materials were then compressed to form sample panels. Prior to the melt-compounding process with PP and NR in the mixer, a pre-mixing of GNP powder with LNR was carried out via ultrasonication for 1-4 h in an ultrasonic instrument (Wiseclean at 75°C, 290W and 50 Hz). The complete mixing process of PP, NR and pre-mixture of LNR/GNP was conducted for 13 min at 180°C and a rotating screw speed of 100 rpm. The matrix of PP/NR/LNR was fixed at a composition ratio of 70:20:10, while the weight fraction of GNP powders was varied at 0.5, 1.0, 1.5 and 2.0 wt.%. After compounding, a compression molding was performed on the mixed materials via hot/cold pressing (LP50, LABTECH Engineering Company LTD) to form composite panels for further characterization. The pressure and pressing period applied for compression molding was 6.9 MPa and 18 min, respectively.

### Characterization

The mechanical properties of the investigated samples (PP/NR/LNR blend and GNP-reinforced nanocomposites) were conducted by tensile, three-point flexural and impact testings of ASTM D638-03, D790-03 and D256-05, respectively. For tensile testing, dumbell-shaped specimens with a thickness of 3 mm were used, whereas the flexural-tested specimens have a dimension of 3.0 mm thickness, 127.0 mm length and 12.7 mm width. Both tensile and flexural measurements were performed using a universal testing machine (model Testometric M350-10CT) with a load cell of 5 kN. The notched samples with a dimension of 64.0 mm x 12.7 mm x 3.0 mm (length x width x thick) were used to carry out the Izod impact testing using a Ray-Ran Universal Pendulum Impact System, with a velocity of 3.46 ms^-1^, a load weight of 0.452 kg and calibration energy of 2.765 J. At least five specimens were tested for each formulation to obtain average values in all mechanical tests.

Wide angle X-ray diffraction (XRD) analysis was performed with a D8 Advance diffractometer with a CuK_α_ radition (λ = 1.54056 Å). The operation of the generator was carried out at 40 kV and 30 mA. The scanning angle used was from 25 to 28° and a scanning rate of 2°/min was applied.

Thermogravimetric analysis (TGA) and differential scanning calorimetry (DSC) were performed using Mettler Toledo TGA/SDTA851^e^ and DSC 882^e^, over the temperature range from 30°C to 600°C and from 30°C to 250°C, respectively. Both testings were run on an approximately 10-15 mg samples at a heating rate of 10°C/min under atmospheric of nitrogen gas flow condition.

Thermal conductivity was conducted via a laser flash method using a thermal conductivity analyser (Nanoflash NETZSCH- model LFA 44712-41). Disc-shaped specimens having a diameter of 12.7 mm and a thickness of 1 mm were tested at room temperature. The electrical properties were measured at room temperature with a constant frequency of 1 Hz to 10^6^ Hz and an AC amplitude of 100–3000 mV using an impedance analyser model (Solatron Model 1255). The disc-shaped specimen with 2 mm thickness and 15 mm diameter was placed on the electrode (as the sample holder). Before testing, the specimen was coated with silver paint to provide conductive surface on the specimen and prevent charging. The measurement of electrical properties was conducted using a computer software ZPlot and analyzed using a ZView software.

An one-way analysis of variance (ANOVA) with the assistance of Data Analysis ToolPak in Ms Excel was performed to statistically (at the 5% significance level) compare the effects of ultrasonic treatment hours and GNP weight fractions on the measured data of mechanical properties.

## Results and discussion

### Mechanical properties in tension

**Fig 1:**
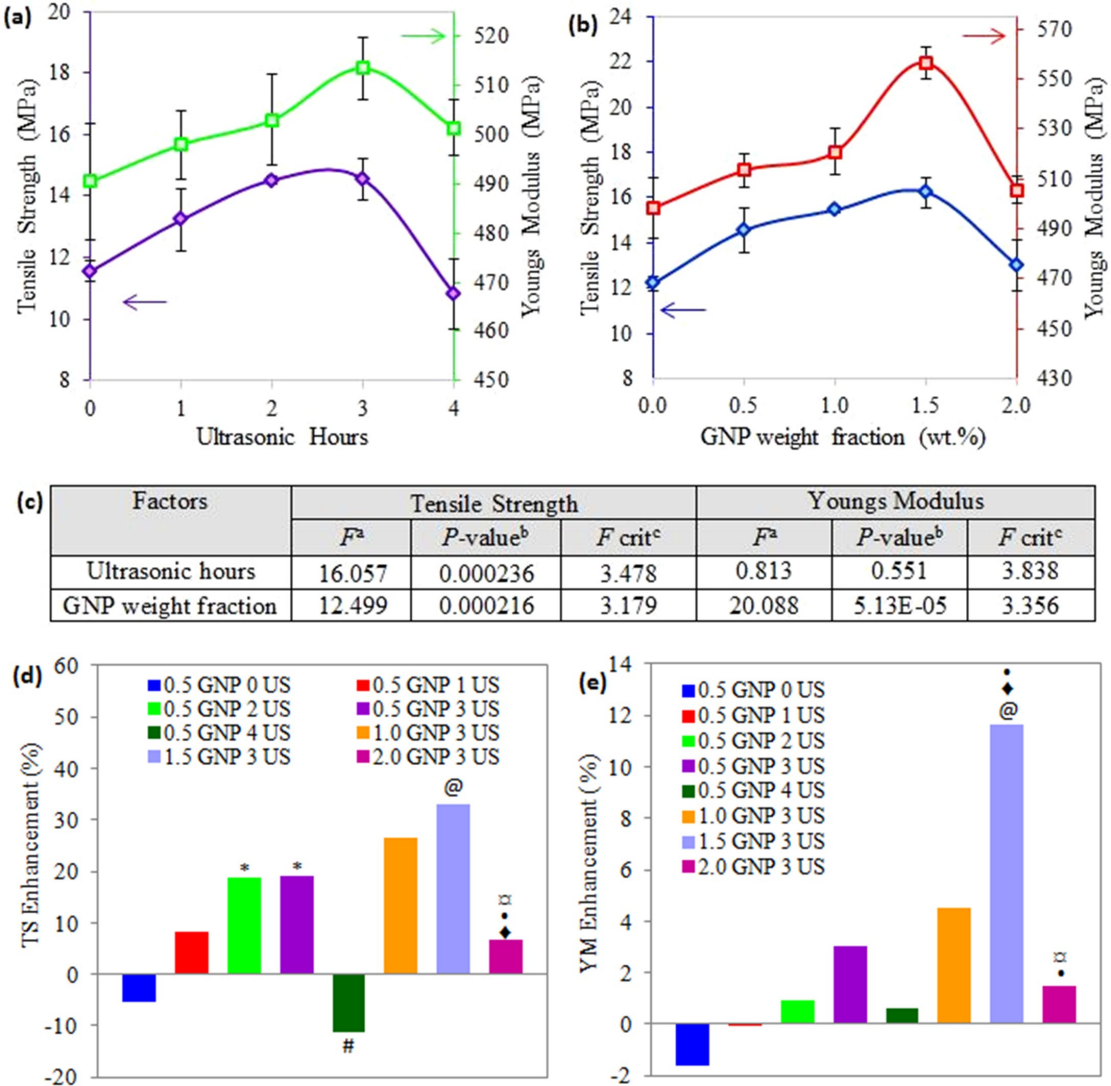
Tensile strength (TS) and Youngs Modulus of TPNR/GNP composites (a) treated with different ultrasonic (US) hours, (b) at various filler weight fractions, (c) ANOVA analysis for both factors in tensile properties, (d) TS enhancement and (e) YM enhancement of investigated composites compared with pure TPNR. Note: ^a^*F* value = mean between groups variance/ mean within group variance; ^b^Probability from 0 to 1; ^c^critical *F* value based on F distribution. Groups with a significant difference (p < 0.05) compared to (i) TPNR/GNP composite without US assistance (0 hour) and assisted with 3 hours US are indicated by * and # respectively; (ii) TPNR composites without GNP, incorporated with 0.5 wt.% GNP, 1.0 wt.% GNP and 1.5 wt.% GNP are indicated by @, ♦, • and ¤ symbols, respectively.

Fig 1 shows the effect of ultrasonication time and GNP weight fractions on the tensile properties of composites. From the investigation on ultrasonic treatment and time, it was clearly observed that the ultrasonication provided at an appropriate time has a positive influence on tensile strength and Youngs modulus, as illustrated in Fig 1(A). The tensile properties achieved the maximum level at 3 hours ultrasonic treatment of LNR/GNP pre-mixure, i.e. each tensile strength and Youngs modulus increased by 25.8% and 4.7% (significant increase as shown by “*” symbol) as compared to those nanocomposites without ultrasonication. Subsequently, a downward trend (significant decrement as compared to 3 hours US indicated by “#” symbol) was observed when ultrasonication time was further increased to 4 hours. This earlier (increasing) trend is attributed to the dispersion of GNP and tendency of GNP to be wrinkled with the help of sonication. Moreover, this wrinkled GNP may act as spring elements in-plane level as a result of a strong GNP/matrix interface, thereby improving the tensile properties [8]. In chemistry aspect, the incorporation of GNP powder into LNR and the pre-mixture with the assistance of ultrasonic treatment before compounding in the internal mixer could promote the chemical reaction between LNR and GNP, and induce better interaction with the polymeric matrix. LNR with some active terminals, such as hydroxyl (-OH), carbonyl (C = O) and epoxy group [11], are believed to improve the GNP filler-matrix (thermoplastic natural rubber) compatibilization. This can be achieved by interaction with hydroxyl and carbonyl groups at the GNP surface [12, 13]. The latter deterioration of tensile properties was related to reagglomeration or restacking of GNP layers in which the reaggregation was driven by the Van der Waals attraction between the graphene layers during the prolonged period of ultrasonic vibration [14]. Another reason is that the residual stresses developed during this sonication process are as high as causing the fracture of the GNP layers [8]. This result was similarly in agreement with previous study that reported a slight decline in tensile strength of nanocomposite polylactic acid (PLA)/LNR filled nickel zinc ferrite after optimum period of ultrasonication, i.e. 1 hour in that particular case [7].

In 3 hours of ultrasonic treatment, the tensile properties displayed an increasing trend prior to decreasing trend as the GNP increased from 0 to 2 wt.% (Fig 1(B)). Compared to the neat TPNR matrix (0 wt.% GNP), the nanocomposite with 1.5 wt.% GNP exhibited the highest tensile strength (16 MPa) and Youngs modulus (556 MPa), which increased by about 33.0% and 11.7%, respectively. These increments are statistically significant, as represented by “@” symbol in Fig 1(D) and 1(E). The enhancement in tensile properties is ascribed to the significant stiffening effect of GNP in matrix as well as the synergistic roles of homogeneous dispersion of GNP in LNR (within matrix) and strong interfacial adhesion, which provides effective load transfer from the matrix to GNP fillers [15]. However, as the weight fraction of GNP was further increased to 2.0%, the tensile strength and modulus of the TPNR/GNP nanocomposites were conversely decreased to the values near to neat TPNR. The formation of GNP agglomerates (due to π-π interaction between the graphene layers) or debonding of TPNR/GNP interface (due to the weak Van der Waals force between polymer and graphene) or both are responsible for the reduction in tensile properties [16, 17].

In Fig 1(C), the effects of ultrasonic hours and GNP weight fractions are significant for the tensile strength. Meanwhile, for the Youngs modulus, only the GNP loadings showed a significant impact, but it was not significantly related to the time of ultrasonic vibration. These are proven from the analysis of variance by F-tests at a confidence level of 95%, where *P*-values are less than the significance level (0.05) and the *F* values are greater than *F* critical for both the ultrasonic time (on tensile strength only) and GNP weight fractions (on tensile strength and modulus). Overall, by taking neat TPNR as a control sample, the nanocomposites without ultrasonic treatment exhibited reduced tensile strength and Youngs modulus, as represented in the blue colour bar (0.5 GNP 0 US) in Fig 1(D) and 1(E). Meanwhile, the nanocomposites containing 1.5 wt.% assisted with 3 hours ultrasonication (1.5 GNP 3 US) displayed a simultaneous enhancement in tensile strength and modulus with the greatest percentage increase.

### Mechanical properties in flexure (three-point bending)

**Fig 2:**
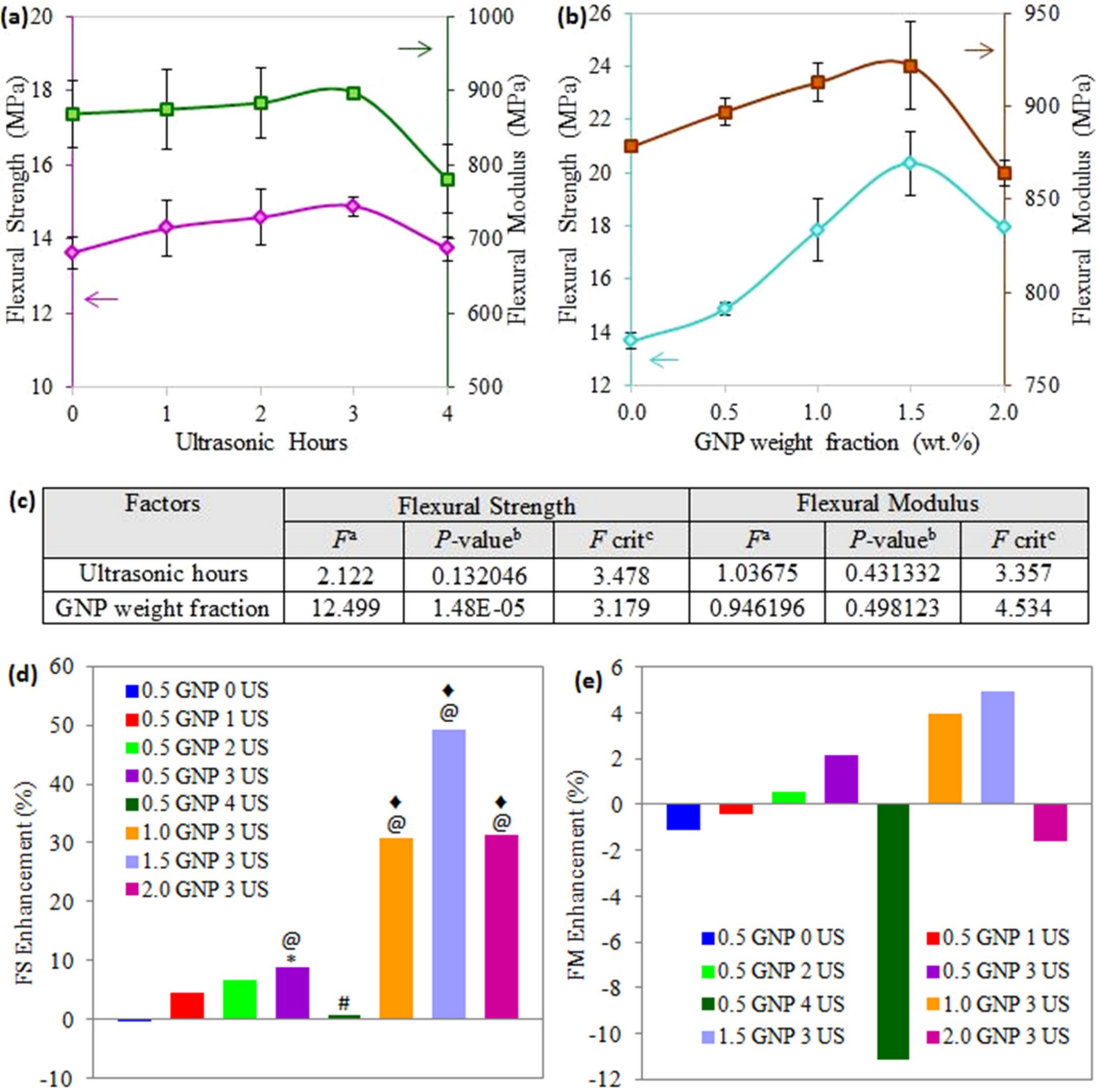
Flexural strength (FS) and flexural modulus (FM) of TPNR/GNP composites (a) treated with different ultrasonic (US) hours, (b) at various filler weight fractions, (c) ANOVA analysis for both factors in flexural properties, (d) FS enhancement and (e) YM enhancement of investigated composites compared with pure TPNR. Note: ^a^*F* ratio = mean between groups variance/ mean within group variance; ^b^Probability from 0 to 1; ^c^critical *F*-value based on F distribution. Groups with a significant difference (p < 0.05) compared to (i) TPNR/GNP composite without US assistance (0 hour) and assisted with 3 hours US are indicated by * and # respectively; (ii) TPNR composites without GNP and incorporated with 0.5 wt.% GNP are indicated by @ and ♦ symbols, respectively.

The influence of ultrasonic time and GNP weight fractions on flexural properties is demonstrated in Fig 2. As compared to tensile results (Fig 1), flexural strength and modulus (Fig 2(A) and 2(B)) showed similar trends of increasing at first to achieve the highest points followed by declining, but with lower changes rate. For the GNP-filled nanocomposite without pre-mixture (LNR/GNP) ultrasonication, flexural strength and modulus were approximately 14 MPa and 869 MPa, respectively. Under ultrasonic application, the sonication time was increased from 1 to 3 hours, the flexural properties increased gradually up to 3.3% (strength) and 9.1% (modulus) as compared to those without sonication. Further extension of the sonication vibration reduced the flexural strength (by 13.0%) and modulus (by 7.7%). Similar to the tensile properties, the nanocomposites reinforced with 1.5 wt.% GNP exhibited flexure mechanical properties at the optimum level, which improved about 7 MPa (by ~49.1%, shown by “@” symbol) and 44 MPa (by ~5.0%) compared to neat TPNR. As shown in Fig 2(B), the higher GNP weight fractions led to a consequent decrease in flexure performance. This behaviour is common in composites incorporated with nano-scale fillers and is probably correlated to dispersion problems. Aggregates are definitely formed at either high concentrations of nanofiller or improper ultrasonic vibration times. This is due to the decreased dispersion degree of GNP nanofiller in the nanocomposites, in which the aggregates could act as stress concentrators to reduce the flexural performance [8].

**Fig 3:**
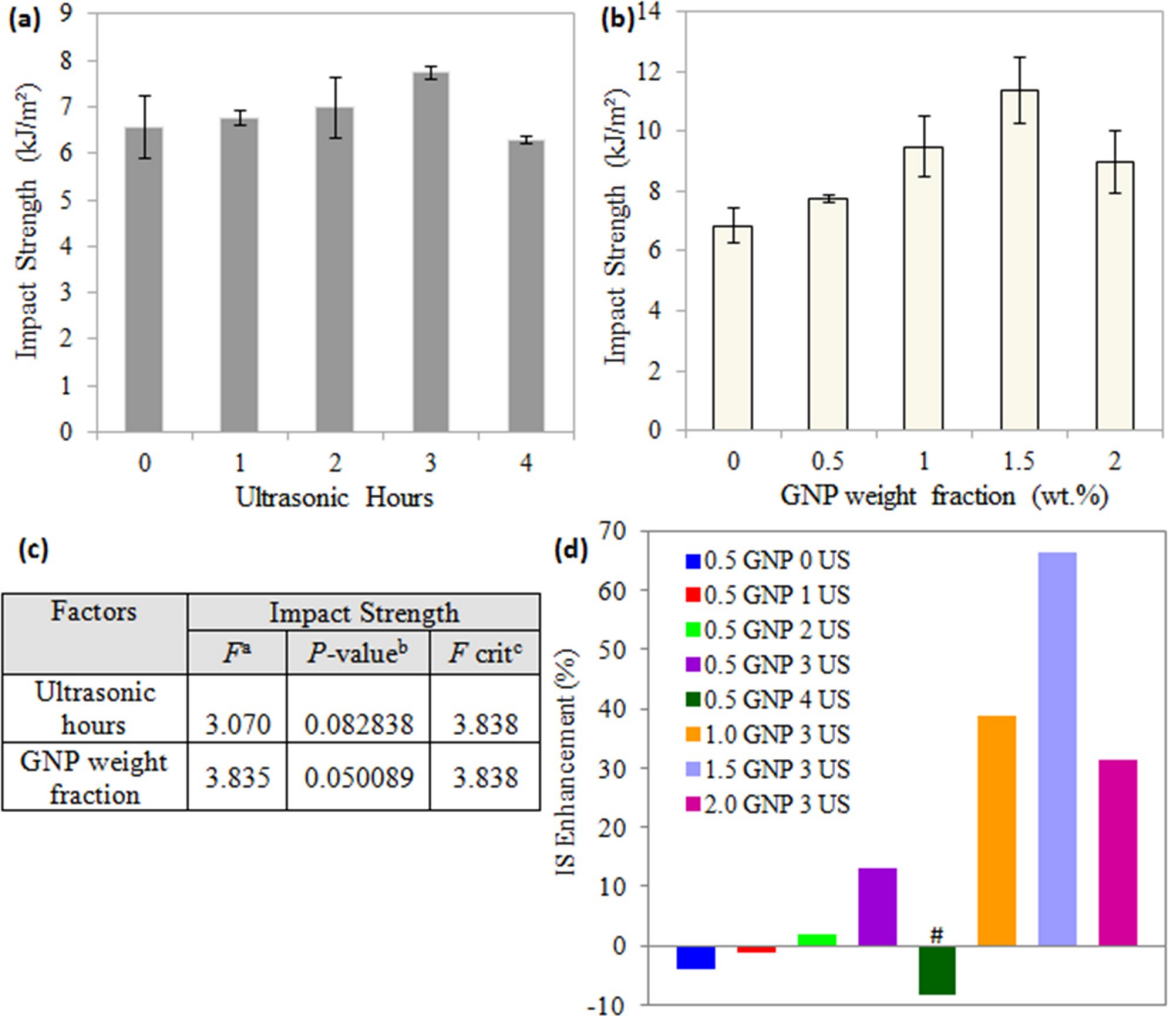
Impact strength (IS) of TPNR/GNP composites (a) treated with different ultrasonic (US) hours, (b) at various filler weight fractions, (c) ANOVA analysis for both factors in impact properties, and (d) IS enhancement compared with pure TPNR. Note: ^a^*F* value = mean between groups variance/ mean within group variance; ^b^Probability from 0 to 1; ^c^critical *F* value based on F distribution. Groups with a significant difference (p < 0.05) compared to TPNR/GNP composite assisted with 3 hours US is indicated by # symbol.

In statistical analysis (Fig 2(C)), the effect of the ultrasonic times is insignificant for the above properties as observing the *p*-values are greater than 0.05 and the *F* values are less than the *F* critical values. On the other hand, it is observed that the incorporation of GNP at different weight fractions has a significant effect on flexural strength, for instance, the remarkable changes are represented by “@” and “♦” symbols based on TPNR composites 0 wt.% and 0.5 wt.% GNP, respectively (Fig 3(D)); but effect on the flexural modulus is small (insignificant).

### Izod impact properties

The measurement of impact strength is likely related to the changes in the energy absorbing mechanisms such as increased plastic deformation of the matrix phase along the matrix-filler interface, crack branching caused by disturbance of fillers, bridging of the crack, formation of voids and crazes at the filler edges [18]. Fig 3 displays the impact strength of TPNR/GNP composites treated with different ultrasonic hours and reinforced with various filler weight fractions. In Fig 3(A), from the aspect of sonication time, the impact strength showed a small increase as the ultrasonic treatment was applied up to 3 hours, which was 17.7% higher than that of without sonication (0 hour), followed by 4.4% reduction. Both effects were statistically insignificant (*P*-value (0.082838) > 0.05; *F* values (3.070) < *F* critical (3.838)).

Comparing to neat TPNR (0 wt.% GNP) having an impact strength of 7 MPa, an improvement (13.0 – 66.4%) was seen when 0.5-1.5 wt.% GNP was incorporated, as observed in Fig 3(B). The results indicate that the reinforcement in the TPNR dispersed well and provided sufficient interface to allow the load transfer from the matrix to the filler. In this case, GNP imparted a positive reinforcing effect on the mechanical properties of the entire weight fraction of less than 2.0% to improve the low stiffness and poor impact toughness of PP [15] (the major component in the matrix). The further increasing of GNP weight fractions (2.0 wt.% in this study) had a negative impact on the mechanical performance, i.e. 26.7% reduced as compared to 1.5 wt.% GNP filled nanocomposites. This deterioration in impact strength is considered to be due to the poorer interfacial interaction occurred between the GNP and the TPNR matrix, which actes as an obstacle for achieving effective stress transfer at the interfaces. The presence of GNP, especially in the case of exceeding the nanofiller loadings, can cause the immobilization of the macromolecular chains, thereby increasing the brittleness (reduced impact strength) of PP-based matrix [19]. Generally, this increment and decrement seem to achieve the significance level (0.05), as the *P*-value (0.050089) is close to 0.05 (Fig 3(C)).

The overall impact strength properties changes for the investigated composites are compared in Fig 3(D). In comparison to neat TPNR, the sonication at appropriate times was necessary in incorporating GNP fillers as only 2 and 3 hours of ultrasonic vibrations were shown to give improvement in impact performance, while longer times of ultrasonication (4 hours) resulted in a consequent decrease in impact toughness due to the re-agglomeration of GNP fillers and this reduction is significant (“#” symbol) as compared to 3 hours (optimized period).

### X-ray diffraction (XRD)

**Fig 4:**
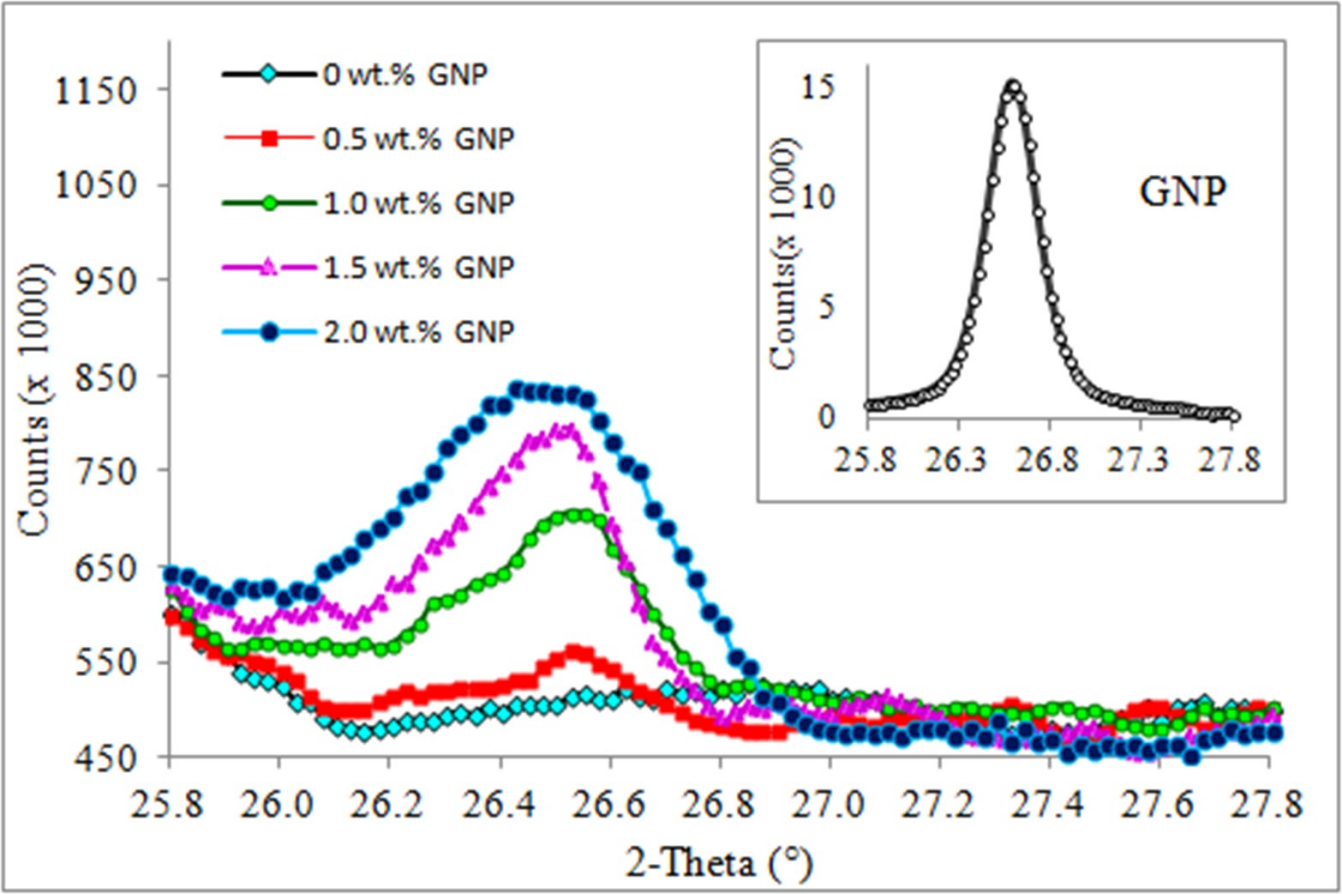
Effect of GNP weight fractions on the nanofillers dispersion in within TPNR matrix [Inset: XRD pattern for pristine GNP].

Fig 4 shows the XRD patterns for pristine GNP and TPNR/GNP nanocomposites with respect to GNP weight fractions. Before compounding, the pristine GNP displayed the characteristic peak at 26.5° with an interlayer spacing of 3.36 Å, which is corresponding to graphitic (002) plane. It is obviously seen that there is negligible difference at this diffraction angle for all TPNR/GNP nanocomposites. This implies that the presence of GNP in the nanocomposites may not have been fundamentally exfoliated but the improvement of nanocomposite properties might be ascribed to the homogenous distribution of GNP in the TPNR matrix. These observation and explanation were reported in literature [20]. In this study, the GNP nanocomposites with different nanofiller weight fractions showed the variation of XRD peak in terms of peak intensity and breadth. As the weight fractions of the added GNP increased, the intensity of the diffraction peak became higher and broaden accordingly. The lower the GNP weight fractions, the easier the platelet layers of GNP fillers are dispersed. It is reasonably proposed that the 0.5 wt.% GNP nanocomposites exhibited a partially exfoliated structure, whereas the intercalation and stacking of GNP layers occurred at higher GNP weight fractions.

### Thermal stability of nanocomposites

**Fig 5:**
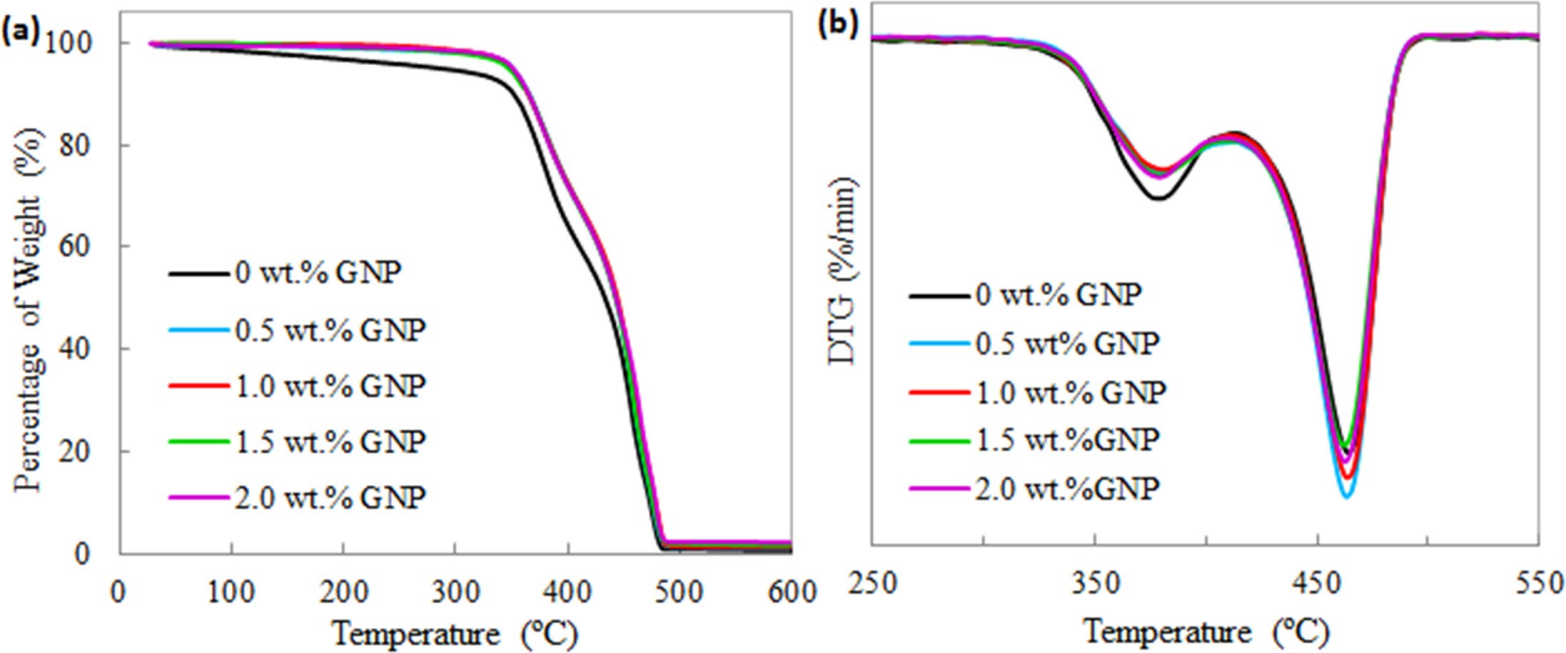
(a) TGA and (b) derivative thermogravimetry (DTG) curves of the neat TPNR sample and TPNR/GNP with different nanofiller weight fractions.

Fig 5 demonstrates the TGA curves of the TPNR sample and TPNR nanocomposites reinforced with various GNP weight fractions. The thermal stabilities of TPNR and TPNR/GNP nanocomposites including decomposition temperature, residues after decomposition and integral procedure decomposition temperature (IPDT) are described in Table 1. In Fig 5(A), the weight loss of the neat TPNR occurred in three steps. The first step started from 100 to 320°C, which is due to the evaporation of bound water molecules in rubber [21]. Interestingly, this stage of weight loss was not observed for TPNR nanocomposites upon the reinforcement of GNP. The following stages of weight loss initiated at 330°C and ended at 480°C, which similar behaviours are observed for both TPNR sample and TPNR/GNP nanocomposites. This suggests that the thermal behaviour of the samples was manipulated by the TPNR which acted as a matrix. The major degradation peaks of the neat TPNR happened at 379 and 464°C. The earlier stage within the temperature range of 340-410°C is related to the decomposition of NR component [22]. The increase of T_1_ indicates the improvement of thermal stability of TPNR by the presence of GNP promoted barrier effect in the TPNR matrix [23]. Meanwhile, the latter stage of weight loss could be attributed to either the continuous degradation of NR as has been reported in literature [22] that NR decomposed in two steps, or the thermal degradation of the polymer as in agreement with Jeske et al. [24] showing maximum mass loss rate of PP at 462°C. These degradations occurred via oxidation and chain scission [21]. However, the incorporation of GNP at low weight fractions slightly influenced the rate of the decomposition as observed by the shiftment of the TGA curves throughout the heating temperature. In general, except for 2.0 wt.% GNP nanocomposites, the nanocomposites filled with 0.5 – 1.5 wt.% GNP exhibited T_1_ at higher temperature but T_2_ at lower temperature as compared to the neat TPNR. The temperature differences between T_1_ and T_2_ in nanocomposites were observed to be smaller (83°C) in comparison to that of the neat TPNR (85°C). This probably implies that the incorporation of GNP in TPNR matrix had a good blending and interfacial interaction.

**Table 1:** Thermal stabilities of TPNR and TPNR/GNP nanocomposites obtained from TGA.

	T_1_ (°C)	T_2_ (°C)	ΔT = T_2_ - T_1_ (°C)	Residues (%)	IPDT (°C)
TPNR (0 wt.% GNP)	379	464	85	0.6	416
0.5 wt.% GNP nanocomposites	380	463	83	0.6	420
1.0 wt.% GNP nanocomposites	380	463	83	1.2	437
1.5 wt.% GNP nanocomposites	380	463	83	1.6	436
2.0 wt.% GNP nanocomposites	378	460	83	2.2	444

Note: T_1_; first decomposition temperature peak, T_2_; second decomposition temperature peak, ΔT; temperature difference, IPDT; integral procedure decomposition temperature

After heating close to 600°C, the residues left upon the decomposition were approximately 0.8-2.3%. The TPNR/0.5 wt.% GNP nanocomposites exhibited the smallest amount of residues (0.6%) and it was similar to neat TPNR sample. This situation because of the further breakdown of the neat polymers into gaseous products at higher temperature beyond the decomposition temperature [25]. Further increasing the GNP weight fractions, the amount of residues increased accordingly up to 1.2, 1.6 and 2.2% for 1.0, 1.5 and 2.0 wt.%, respectively. The residue amounts were about the weight fractions of GNP which clearly means that the GNP was not burnt off at the temperatures below 600°C as a result of the inert atmosphere [26]. This is similar to the research study of Liang et al. [27] who stated the residues found in PP/GNP composites were mainly the GNP.

The IPDT calculated in Table 1 is used to estimate the inherent thermal stability of polymeric materials, using the following equation: IPDT (°C) = A*K*(T_f_ -T_i_) + T_i_, where A* = (S_1_ + S_2_) / (S_1_ + S_2_ + S_3_) and K* = (S_1_ + S_2_)/ S_1_. In which T_f_ is the final testing temperature, T_i_ is the initial testing temperature, S_1_ is the area under the TGA curves (above the minimum residue level), S_2_ is the area below the minimum residue level of the TGA curve, and S_3_ is the area above the TGA curves. From Table 1, the IPDT value of the neat TPNR was 416°C, and this value increased gradually when GNP was incorporated with the increase of GNP weight fractions (only by 1-7%). This suggests that the TPNR/GNP nanocomposites were more thermally stable than that of the neat TPNR matrix. This trend is agreed by Yadav et al. [28] who studied on carboxymethyl cellulose/graphene oxide nanocomposite film.

### Melting, enthalpy and crystallinity properties of nanocomposites

**Fig 6:**
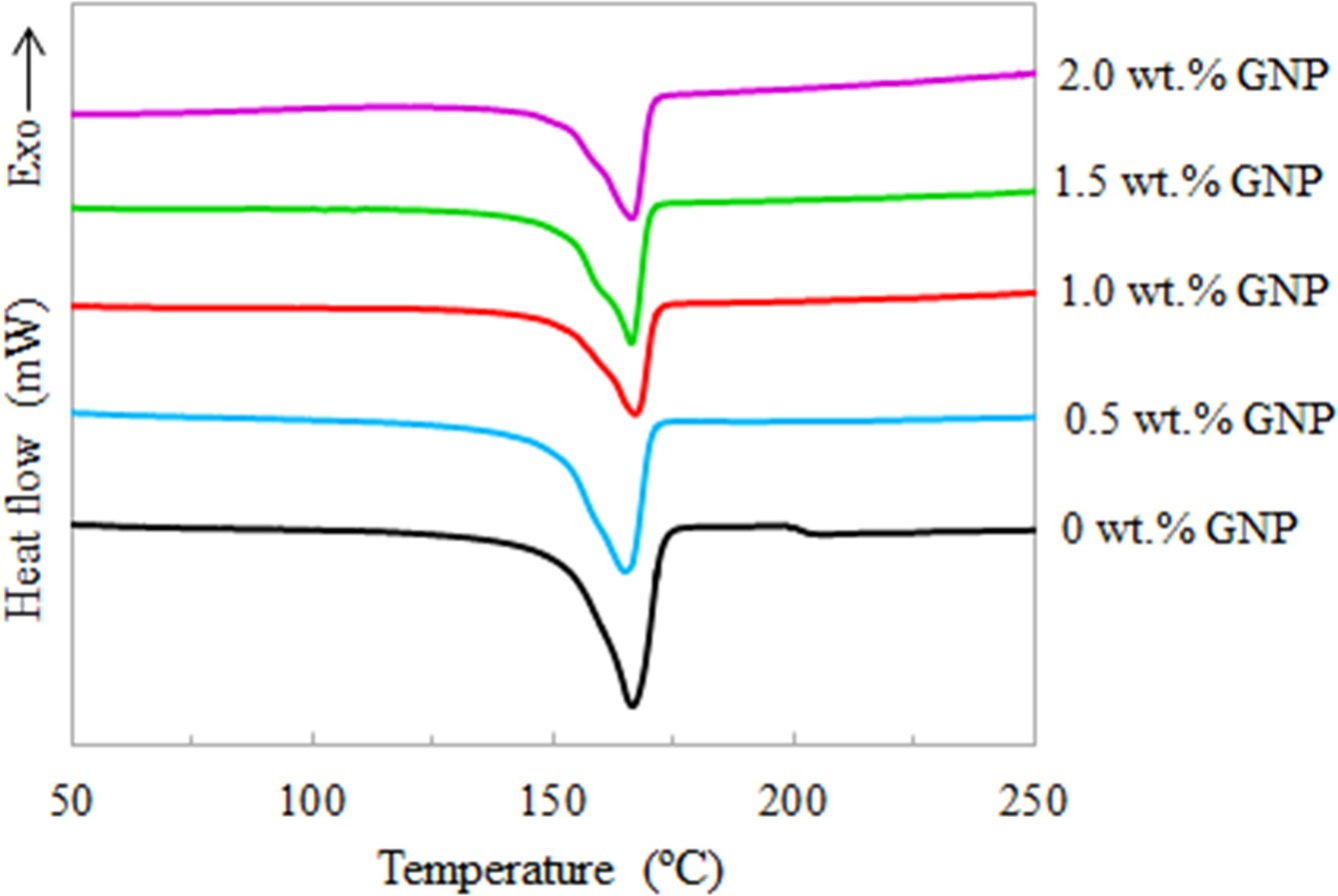
DSC thermograms of the neat TPNR sample and TPNR/GNP with different nanofiller weight fractions.

Fig 6 displays DSC thermograms of the neat TPNR sample and TPNR/GNP nanocomposites during heating (melting) process. The thermal property characteristics of the investigated samples are listed in Table 2. It can be clearly observed that the melting process of the samples occurred at around the same temperature range, from 150 to 170°C. The position of T_onset_, T_m_ and T_endset_ showed insignificant changes with respect to the GNP weight fractions incorporated in the TPNR. As compared to neat TPNR (ΔH_m_ = 64.17 J/g), it is found that this value was higher for the TPNR/GNP nanocomposites (ΔH_m_ = ~ 67 to 71 J/g) with an exclusion of 1.5 wt.% GNP (ΔH_m_ = 59.35 J/g). Since the enthalpy of melting has been used to obtain the crystallinity (χ_c_), the changes in χ_c_ were similar as the ΔH_m_. Generally, there are two types of interpretations to explain the effects of GNP on the crystallization behaviour of TPNR. First, the incorporation of GNP can induce the heterogeneous nucleation of GNP and promote a consequent crystallization of TPNR. Second, GNP can restrict the movement of TPNR chains and inhibit the crystallization as a result of the increased viscosity of the samples [29]. In this study, the addition of GNP in nanocomposites, irrespective of GNP weight fractions, has resulted an improvement but inconsistent trend in χ_c_. This first type of effect is dominant. The inconsistent trend might be due to the very small amount of GNP (0.5 – 2.0 wt.%) in the nanocomposite system and the variation of the GNP weight fractions in each nanocomposite was just about 0.5 wt.%. In which this is believed to be strongly correlated to the dispersion of GNP in nanocomposites.

**Table 2:** Thermal property characteristics of TPNR and TPNR/GNP nanocomposites obtained from DSC.

Samples	T_onset_ (°C)	T_m_ (°C)	T_endset_ (°C)	ΔH_m_ (J/g)	χ_c_ (%)
TPNR (0 wt.% GNP)	156	166	172	64.17	43.86
0.5 wt.% GNP nanocomposites	154	167	170	70.81	50.95
1.0 wt.% GNP nanocomposites	156	167	171	67.89	51.56
1.5 wt.% GNP nanocomposites	155	166	170	59.35	47.70
2.0 wt.% GNP nanocomposites	154	166	170	69.66	59.52

Note: T_onset_; onset temperature of melting, T_m_; Melting Point, T_endset_; endset temperature of melting; ΔH_m_; Enthalpy of Melting, and χ_c_; Crystallinity

### Thermal conductivity of nanocomposites

**Fig 7:**
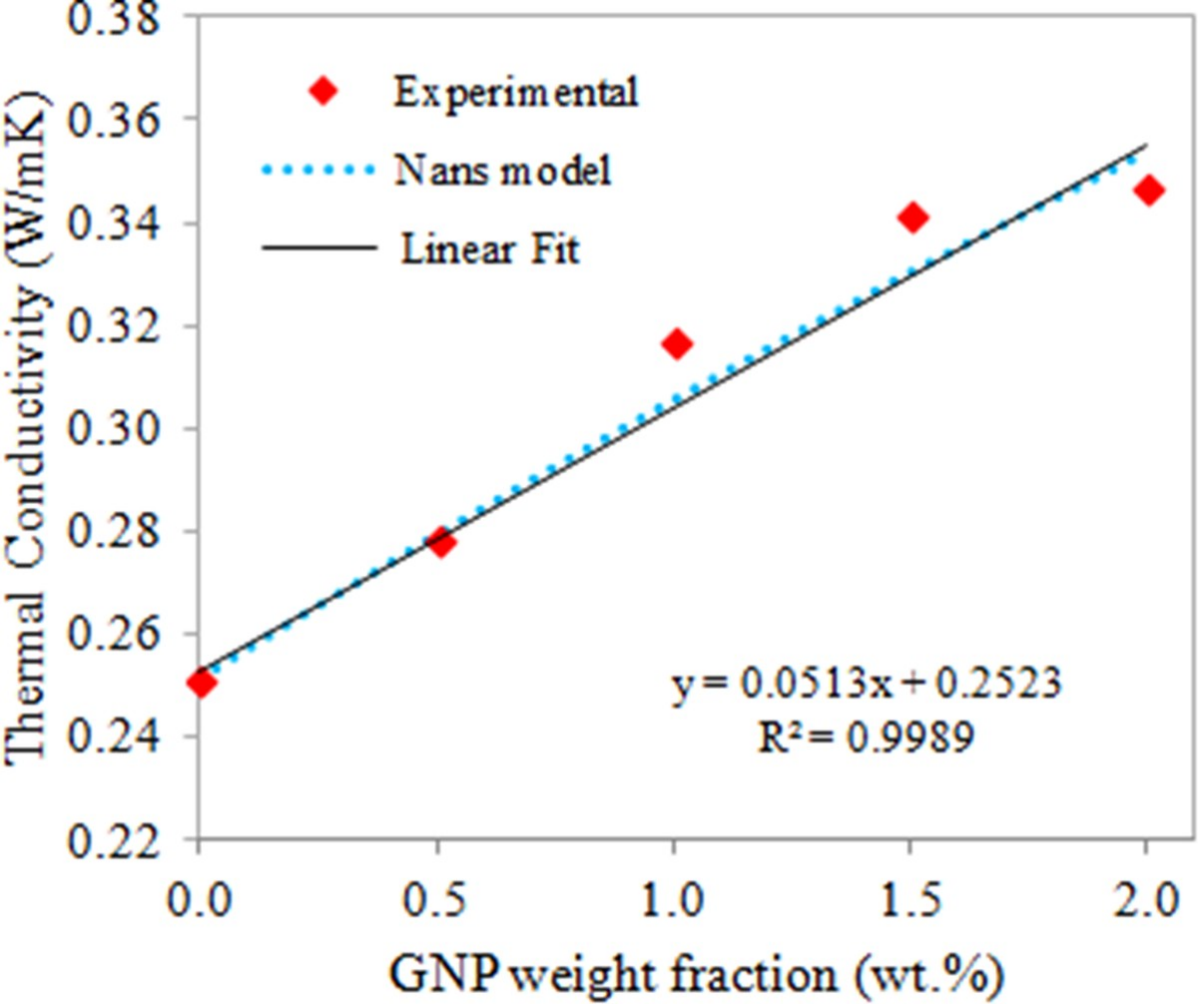
Thermal conductivity of the neat TPNR sample and TPNR/GNP with different nanofiller weight fractions.

As well-established, thermal conductivity of composites is greatly dependent upon the particle dimension and shape, alignment and curliness of particles, particle concentration, state of dispesion, as well as the thermal interface resistance between the matrix and the nanofillers [30]. The thermal conductivity of the samples versus GNP weight fractions is portrayed in Fig 7. It is evident that the experimental thermal conductivity increased steadily as the nanofiller concentrations increased. The relationship between the increment of thermal conductivity and the GNP weight fractions is shown as a nearly perfect linear relationship with the equation of y = 0.0513x + 0.2523, R^2^ = 0.9989. The thermal resistance between GNP and the TPNR matrix as well as the scattering processes on its interface play important roles in the effective heat conduction of the composites. In which the generated heat propagation in GNP-polymer composite is mainly ascribed to acoustic phonons scattering processes [31]. Generally, when GNP is incorporated in a polymer, a large number of GNP-polymer interfaces is produced owing to the extremely high specific surface area of graphene. Consequently, these interfaces would result in phonon scattering and introduce ultrahigh interfacial thermal resistance, thereby causing the difficulty in transferring heat through the interface [32]. Specifically in this TPNR/GNP nanocomposites, GNP acted as a highly thermal conductive channel or heat transfer media, while the use of LNR aided to promote interaction and bonding between GNP and the molecular chains of the polymer matrix, which subsequently facilitate the phonon transfer from the GNP to the polymer and vice versa. In this case, the experimental thermal conductivity increased with the increasing GNP weight fractions and there was no percolation threshold phenomenon (no critical loading, which is a loading of which the conductivity is improved remarkably) shown in thermally conductive composites.

**Fig 8:**
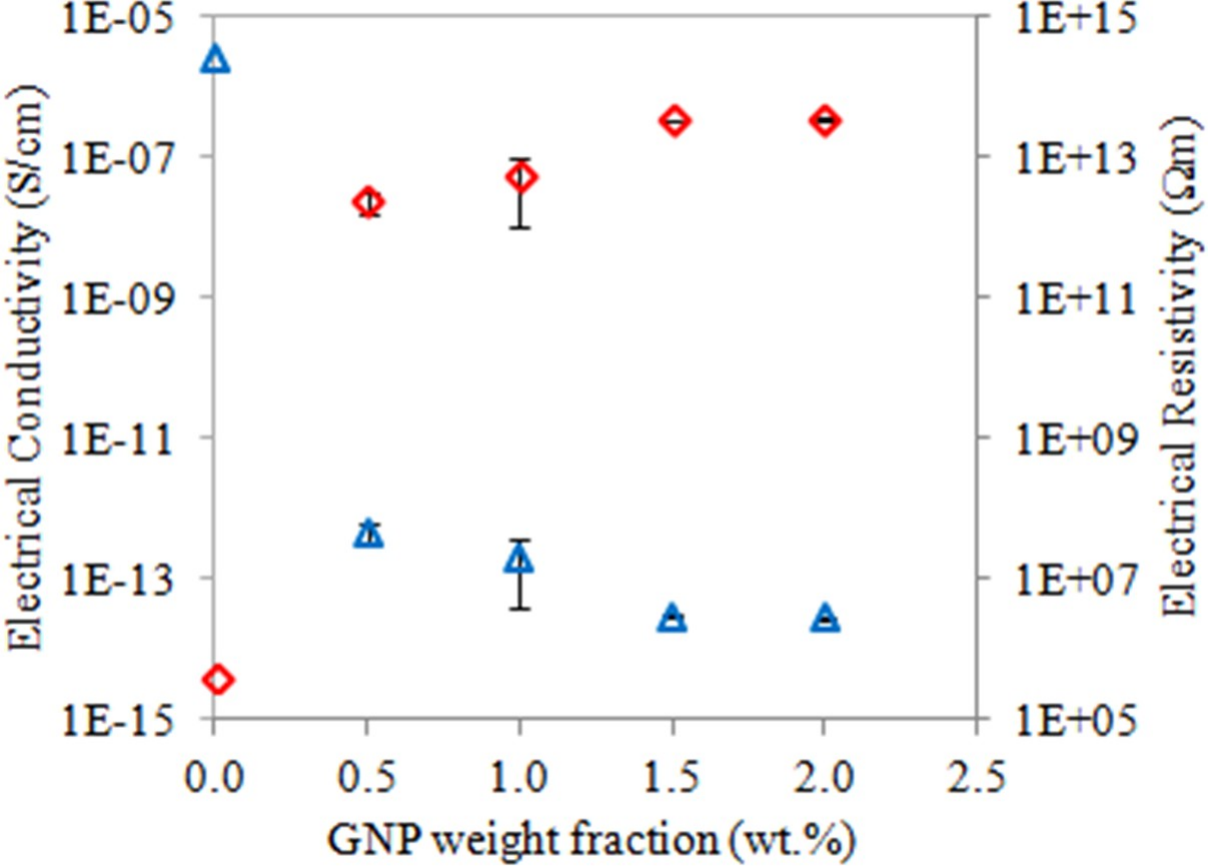
Electrical conductivity and resistivity of the neat TPNR sample and TPNR/GNP with different nanofiller weight fractions.

The behaviour of thermal conductivity in this study was in agreement with Nan’s model [33, 34] as illustrated by the dotted line in Fig 8, which shows close overlapping with linear line, assuming that interfacial thermal resistance (ITR) existed between the uniformly dispersed nanofillers in the insulating polymer matrix. The calculated ITR of this nanocomposite system from the Nans model was ~ 10^-7^ m^2^K/W. This ITR value is consistent with the recently published values in GNP/boron-nitride/epoxy [33] system (~ 10^-7^ m^2^K/W), graphene-coated copper/water [35] system (0.8 x 10^-7^ m^2^K/W) and GNP/epoxy [36] system (0.1 x 10 ^-7^ m^2^K/W).

### Electrical properties of nanocomposites

Fig 8 shows the electrical conductivity and resistivity of the nanocomposite samples reinforced with various GNP weight fractions. The electrical conductivity of the neat TPNR is virtually null (3.8 x 10^-15^ S cm^-1^), which affirms the insulating characteristic of this material. Nanocomposites TPNR/GNP containing 0.5 wt.% exhibited a higher electrical conductivity (here increased by ~ 7 orders of magnitude) reaching of 10^-7^-10^-8^ S/cm, as compared to TPNR blend. This indicates that the electrically conductive nanocomposites show the percolation phenomenon where a conductive network has been formed between the conductive GNP. The conductivity increased with much lower rate with increasing the weight fractions of GNP filler. As expected, the electrical conductivity trend was opposite of the electrical resistivity where the electrical resistivity of TPNR was shown at 2.6 x 10^14^ Ωm and this value increased greatly with the presence of 0.5 wt.% and higher weight fractions of GNP. At GNP weight fraction of 0.5 wt.%, the GNP particles approach each other and this significantly improves the interactions between the neighbouring GNP fillers. The interconnected GNP particles induce path for electrical conduction, thereby showing the comparatively low resistance [37]. The electrical conductivities of the samples obtained in this work are consistent with the findings published in other works related to GNP [38, 39].

## Conclusions

GNP reinforced TPNR conductive nanocomposites were fabricated using internal mixing and compression molding. The effects of ultrasonic pretreatment time of LNR/GNP and low weight fractions of GNP on the TPNR/GNP nanocomposite properties were examined. From mechanical results, it showed that the assistance of ultrasonic premixing of LNR/GNP at 3 hours provided the distribution of GNP particles within the TPNR matrix and resulted in a consequent increase of mechanical properties. The mechanical properties of TPNR/GNP nanocomposites achieved the optimum level at 1.5 wt.% GNP. In thermal aspect, the incorporation of GNP gave a positive effect in thermal stability and conductivity properties. The GNP-filled nanocomposites exhibited an outstanding electrical conductivity as compared to that of conventional elastomeric blend. It can be concluded that the multifunctional TPNR/GNP nanocomposites could be a potential candidate in conductive applications.
